# The effects of methionine administration during the beginning postnatal days on the ovarian structures in adult rats

**DOI:** 10.1002/vms3.750

**Published:** 2022-02-08

**Authors:** Mohamad Naser Nazem, Seyed Morteza Aghamiri, Reza Kheirandish, Zeinab Hakimy

**Affiliations:** ^1^ Department of Basic Science Faculty of Veterinary Medicine Shahid Bahonar University of Kerman Kerman Iran; ^2^ Department of Clinical Science Faculty of Veterinary Medicine Shahid Bahonar University of Kerman Kerman Iran; ^3^ Department of Pathobiology Faculty of Veterinary Medicine Shahid Bahonar University of Kerman Kerman Iran

**Keywords:** corpus luteum, histomorphometry, histopathology, methionine, ovarian follicles

## Abstract

**Objectives:**

Methionine is known as an essential amino acid in mammals. Consuming excessive amounts of methionine has toxic effects. This study aimed at evaluating the histomorphometric and histopathologic changes of ovaries after methionine administration during follicle formation.

**Material and methods:**

A total of 60 newborn female rats born under similar conditions were selected and randomly assigned into three groups including control, recipients of 50 and 200 mg/kg body weight of methionine for 5 days. On day 120, all 60 female rats were euthanized and the whole left ovary of each animal was taken in order to count the number of primordial, primary, secondary, antral, atretic follicles, as well as corpora lutea and also to conduct histopathologic study.

**Results:**

According to the results, the 50 mg/kg methionine did not significantly change the number of primordial follicles compared to the control group but the 200 mg/kg dose significantly decreased the number of primordial follicles. There were no significant differences between the groups in the number of other types of follicles and also in the number of corpora lutea. There was no histopathological lesion in the groups.

**Conclusions:**

It seems that the high dose of methionine could exacerbate apoptosis of the primordial ovarian follicle during the follicle assembly process. However, the remaining were enough to form later stages of follicles after puberty.

## Graphical abstract

A high dose of methionine could exacerbate apoptosis of the primordial ovarian follicle during the follicle assembly process.

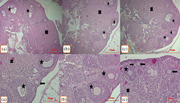

## 1 INTRODUCTION

Primordial follicles development determines the follicular reserve of ovaries and is an important stage in the ovarian organization (Gaytán et al., 2014). Follicle formation occurs during mid‐gestation in humans and the beginning days after birth in rodents (Gaytán et al., 2014; Tingen et al., 2009). In the early ovaries, the oocytes are surrounded by somatic pre‐granulosa cells and form ovigerous cords. Then, apoptosis occurs in more than half of the oocytes and the remaining is surrounded by a layer of granulosa cells, forming the primordial follicles (Gaytán et al., 2014).

Epidemiological and experimental studies on humans and animals have shown that mothers’ nutrition during different stages of pregnancy could cause permanent changes in the structure, physiology and metabolism of their offspring (Burdge & Lillycrop, 2010; Chmurzynska, 2010). Changes in maternal nutrition affect oocyte maturation, fertility, implantation, embryo and fetal development (Ashworth et al., 2009). The epigenetic changes could be induced by the maternal feeds such as DNA methylation causing a permanent alteration in the offspring phenotype (Burdge et al., 2007). The DNA methylation depends on the availability and supply of methyl group by various amino acids including methionine and compounds such as choline, vitamin B12 and folic acid (Van den Veyver, 2002).

Methionine is an essential sulfuric amino acid necessary to maintain proper growth and development in mammals and plays a critical role in the synthesis of vital molecules including cysteine, carnitine, lecithin, phosphatidylcholine and other phospholipids (Ursini & Pipicelli, 2009). The excess amount of methionine in the diet causes many toxic effects including reduced food intake and growth rate and also, some abnormalities in tissue formation (Koz et al., 2010; Peng & Evenson, 1979).

Studying the number of follicles could provide important information about the ovarian function, especially the relationship between folliculogenesis and the factors regulating it (Tilly, 2003). Considering the adverse effects and apoptosis induction of excess methionine on some organs including adult mammalian skin (Nazem et al., 2016), pup rat brain (Koz et al., 2010) and ovarian tissue (Al‐Alim et al., 2013; Nazem et al., 2017), this study evaluated the effects of methionine on the ovaries during follicle formation at the beginning postnatal days in rats.

## 2 MATERIALS AND METHODS

### 2.1 Animals and housing

A total of 20 adult females (230 ± 10 g) and 10 adult males (250 ±15 g) Wistar rats were used for this study. During the experiment, the animals were kept in plastic cages at a temperature of 22–25°C, 12 h light/dark cycle and had free access to the commercial rodent food (Javaneh Khorasan Co., Mashhad, Iran) (Table 1). Animals were kept for 1 week to be adapted to the environmental conditions.

**TABLE 1:** Analysis of commercial rodent food (Javaneh Khorasan Co.)

Crude protein (%)	20
Fat (%)	4
Crude fibre (%)	4.5
Ash (%)	<10
Moisture (%)	<10
Salt (%)	0.5
Calcium (mg/kg)	10,000
Phosphorus (mg/kg)	7000
Lysine (mg/kg)	11500
Methionine (mg/kg)	3300
Methionine + Cysteine (mg/kg)	6300
Threonine (mg/kg)	7200
Tryptophan (mg/kg)	2500
Energy (kcal/kg)	4100

### 2.2 Experimental design

In each mating cage, one male and two female rats were mated for seven days. After 1 week, male rats were isolated. After the pregnancy period, all female rats gave birth during 5 consecutive days. About 60 newborn female rats after sex determination (according to the distance between the anus and the external genitalia) were randomly divided into three groups of control and two methionine receiving groups (50 and 200 mg/kg body weight). l‐Methionine (Scharlau Co., Spain) solution was prepared by dissolving the mentioned amounts of methionine in 0.1 ml sterile normal saline for intraperitoneal injection (Nazem et al., 2016). In order to ensure the methionine intake, its solution was injected intraperitoneally. The newborn female rats received the solution from the birth for 5 days (Rodríguez et al., 2010). The control group was injected 0.1 ml of normal saline for 5 days.

### 2.3 Histological and histopathological preparation of ovaries

Animals were euthanized on day 120 by intramuscular injection of high dosage of sodium thiopental. In the following, left ovary of each animal was removed from the abdominal cavity. The specimens were fixed in 10% neutral buffered formalin for 10 days, dehydrated in graded anhydrous absolute ethanol and xylol alcohols and then embedded in paraffin blocks. Frozen paraffin blocks were cut into 5 μm sections. One section from each of 10 consecutive sections was selected to be stained with haematoxylin‐eosin for histomorphometric and histopathological studies. Depending on the size of each ovary, 30–38 sections were obtained to analyze.

### 2.4 Statistical analysis

Obtained data were analyzed by SPSS software version 16 (SPSS for Windows, SPSS Inc, Chicago, Illinois). The numbers of ovarian structures including primordial, primary, secondary, antral, atretic follicles and corpora lutea were reported as means ± SE. One‐way analysis of variance (ANOVA) and Tukey's test were used to analyze the data. In this study, *p* < 0.05 was considered as the significant level.

## 3 RESULTS

### 3.1 Histopathologic findings

**FIGURE 1:**
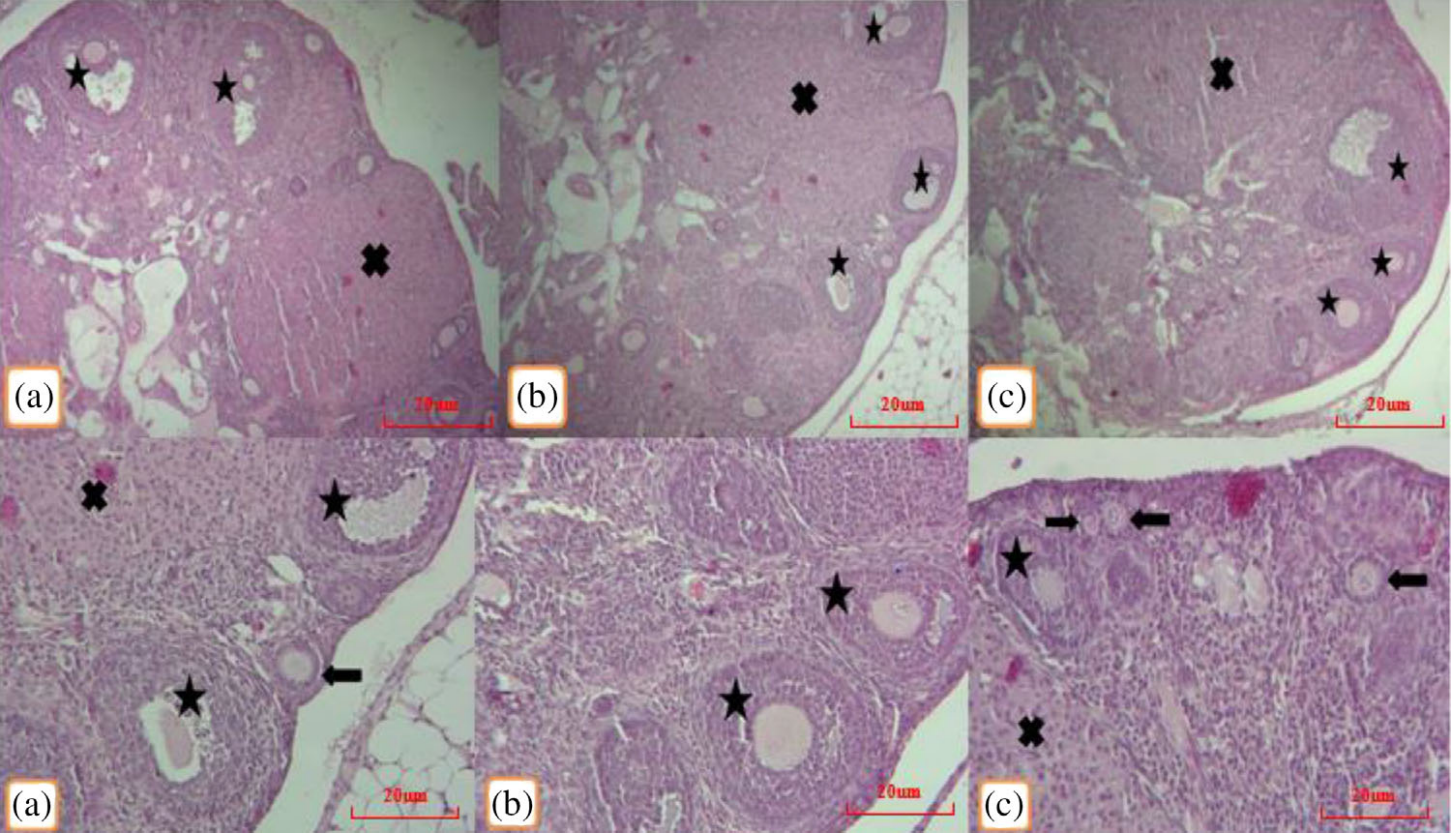
Different follicles including primary follicles (→), antral follicles (*) and corpus luteum (×) are shown in control (a), receiving of 50 (B) and 200 (c) mg/kg methionine (H&E)

**FIGURE 2:**
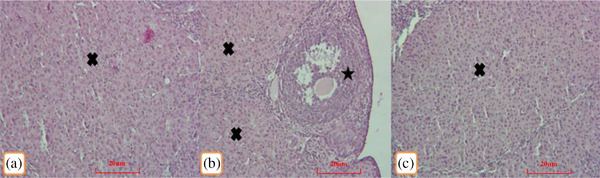
Normal corpus luteum (×) and antral follicles (*) are seen in the control (a), receiving of 50 (b) and 200 (c) mg/kg methionine

Normal ovarian tissues consisting of primary, growing, antral follicles, normal corpora lutea and a few atretic follicles were observed in the control group. In the 50 and 200 mg/kg methionine groups, the ovarian tissue showed normal follicles with no pathological lesions similar to the control group (Figure 1). The shapes of the corpora lutea were similar in all groups, with no signs of congestions or other pathological features. No ovarian atrophy was observed in any of the methionine injected groups compared to the controls (Figure 2).

### 3.2 Histomorphometric findings

**FIGURE 3:**
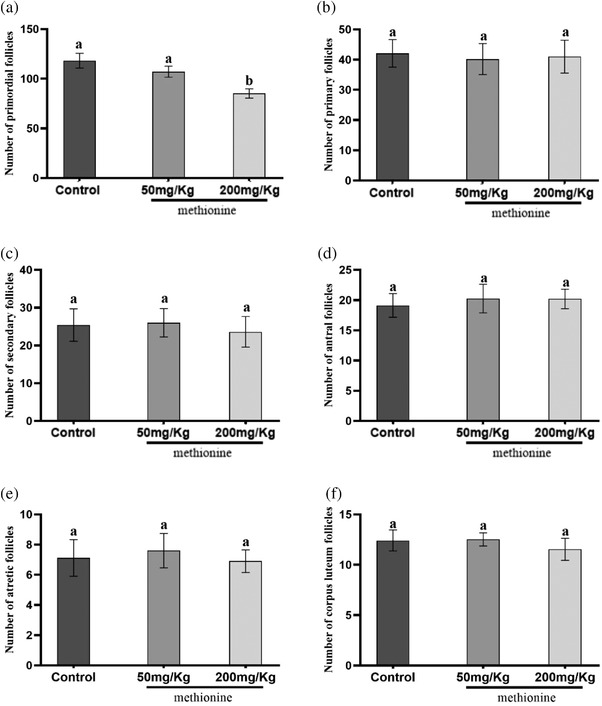
Effects of different treatments on the number (Mean ± SE) of primordial (a), primary (b), secondary (c), antral (d) atretic (e) follicles and corpus luteum (f)

The results showed that the number of primordial follicles in the control group (118.2 ± 12.9) were significantly higher than the group receiving 200 mg/kg methionine (85.27 ± 8.12). In contrast to the primordial follicles, there was no significant difference in the number of primary, secondary, antral and atretic follicles and corpora lutea between the control and experimental groups (Figure 3b–f).

## 4 DISCUSSION

In this study, the effects of methionine administration during follicle assembly on the histomorphometric and histopathologic changes of the ovary in newborn rats was investigated. It is well known that nutritional disorders during pregnancy not only influence the growth and development of the fetus but also affect the future physiologic and metabolic health after maturity (Ashworth et al., 2009; Fleming et al., 2012).

In this study, administration of methionine during follicle formation decreased the number of primordial ovarian follicles although it was significant at the high‐dose group (Figure 3). Unlike this study, no significant difference was found after short‐term administration of methionine in the ovaries of mature rats (Nazem et al., 2017).

Peñagaricano et al. (2013) observed that expression of 72% of important genes decreased in embryos obtained from the methionine‐rich maternal diet which led to differences in the quality of embryos (Peñagaricano et al., 2013) and may have influenced the physiology of offspring (Sinclair et al., 2007). Increasing the levels of methyl donors such as methionine enhances the DNA methylation of many embryonic genes, which, in turn, can suppress gene transcription (Yu et al., 2013).

Homocysteine is a sulphur‐containing amino acid formed by demethylation of methionine (Finkelstein, 1998), and it has been shown that the methionine administration can increase plasma homocysteine concentrations (Koz et al., 2010; Steele et al., 1979). The homocysteine has induced ovarian atrophy with a decrease in growing follicles by infiltration of inflammatory factors such as free radicals into ovarian tissue (Al‐Alim et al., 2013) and even follicular fluid (Berker et al., 2009). There was a relationship between follicular fluid homocysteine content—affected by the body homocysteine metabolism—and human oocyte and the embryo quality (Berker et al., 2009). Based on these studies, the level of homocysteine may be increased in the group receiving 200 mg/kg methionine compared to the control and 50 mg/kg methionine groups at beginning days post‐injection. Because of low volume of the serum, we could not measure the blood homocysteine levels.

An increase in blood homocysteine level may occur following methionine intake (Nazem et al., 2016). The homocysteine leads to toxic and oxidative damages on tissue cells and, subsequently, causes apoptosis due to the breakdown of DNA into smaller fragments (Baydas et al., 2005; Ho et al., 2002; Koz et al., 2010; Kuszczyk et al., 2009). In the present study, exposure to high levels of methionine decreased the primordial follicles. It seems that apoptosis is a possible cause, but not the only one, for primordial follicle loss in the high‐dose methionine group. Although there was a significant decrease in the number of primordial follicles in the high‐dose methionine group (Figure 3a), the results showed that the remaining primordial follicles were enough to form later stages of follicles, especially during early post‐puberty.

## 5 CONCLUSION

The present study showed that the high doses of methionine, as a limiting amino acid, have toxic effects on the number of primordial follicles in the neonatal ovaries of the rats that can be used as an animal model for humans in mid‐gestation. However, the remaining primordial follicles were enough to form later stages of follicles.

## CONFLICT OF INTEREST

The authors declare that they have no conflict of interest.

## ETHICS STATEMENT

This trial was approved by the members of the state commission on animal ethics, Veterinary Faculty of Shahid Bahonar University of Kerman, Kerman, Iran (Approval ID: IR.UK.VETMED.REC.1398.028). The authors confirm that they have followed the recommendations of the European Council Directive (2010/63/EU) of September 22, 2010 for the protection of animals used for scientific purposes.

## AUTHOR CONTRIBUTION

*Conceptualization, methodology and writing—original draft*: Seyed Morteza Aghamiri. *Investigation and methodology*: Reza Kheirandish. *Data curation*: Zeinab Hakimy. *Conceptualization, methodology and writing—review & editing*: Mohamad Naser Nazem. *Conceptualization, methodology and writing—original draft*: Seyed Morteza Aghamiri. *Investigation and histopathology*: Reza Kheirandish. *Data curation*: Zeinab Hakimy. All authors read and approved the final manuscript.

### PEER REVIEW

The peer review history for this article is available at https://publons.com/publon/10.1002/vms3.750

## Data Availability

The data that support the findings of this study are available from the corresponding author upon reasonable request.
